# South-Western Georgia Medical Association

**Published:** 1874-07

**Authors:** 


We are pleased to learn that the physicians of the southwestern portion of Georgia have organized themselves into a medical body, under the name and style of the Southwestern Georgia Medical Association. This organization was effected on the 18th of April last, at Fort Valley, with the following officers chosen for the year, namely: Dr. W. M. Mathews, of Fort Valley, President; Dr. K. P. Moore, of Knoxville, Vice-President; Dr. R. T. Persons, of Fort Valley, Secretary; Dr. W. J. Green, of Fort Valley, Treasurer.

In many portions of our State the population is so sparse as to render it difficult to get up a sufficient membership in any one county for an effective medical organization, but by the association of several counties together, good and useful societies can be formed which are calculated to advance the professional interests of the members and to elevate the profession in general.

We trust we may be able to present to our readers more of the transactions of this Association, and that others of like character with this, and with the Middle Georgia Medical Association, which has been in existence for some time past, will continue to be formed in different sections of the State.

